# Maritoclax and dinaciclib inhibit MCL-1 activity and induce apoptosis in both a MCL-1-dependent and -independent manner

**DOI:** 10.18632/oncotarget.3706

**Published:** 2015-03-30

**Authors:** Shankar Varadarajan, Paramasivan Poornima, Mateus Milani, Krishne Gowda, Shantu Amin, Hong-Gang Wang, Gerald M. Cohen

**Affiliations:** ^1^ Department of Molecular and Clinical Cancer Medicine, University of Liverpool, Liverpool, UK; ^2^ Department of Pharmacology, Pennsylvania State University College of Medicine, Pennsylvania, USA; ^3^ Department of Pediatrics, Pennsylvania State University College of Medicine, Pennsylvania, USA

**Keywords:** dinaciclib, maritoclax, MCL-1, apoptosis, mitochondria

## Abstract

The anti-apoptotic BCL-2 family proteins are important targets for cancer chemotherapy. Specific and potent inhibitors of the BCL-2 family, such as ABT-263 (navitoclax) and ABT-199, are only effective against some members of the BCL-2 family but do not target MCL-1, which is commonly amplified in tumors and associated with chemoresistance. In this report, the selectivity and potency of two putative MCL-1 inhibitors, dinaciclib and maritoclax, were assessed. Although both compounds induced Bax/Bak- and caspase-9-dependent apoptosis, dinaciclib was more potent than maritoclax in downregulating MCL-1 and also in inducing apoptosis. However, the compounds induced apoptosis, even in cells lacking MCL-1, suggesting multiple mechanisms of cell death. Furthermore, maritoclax induced extensive mitochondrial fragmentation, and a Bax/Bak- but MCL-1-independent accumulation of mitochondrial reactive oxygen species (ROS), with an accompanying loss of complexes I and III of the electron transport chain. ROS scavengers, such as MitoQ, could not salvage maritoclax-mediated effects on mitochondrial structure and function. Taken together, our data demonstrate that neither dinaciclib nor maritoclax exclusively target MCL-1. Although dinaciclib is clearly not a specific MCL-1 inhibitor, its ability to rapidly downregulate MCL-1 may be beneficial in many clinical settings, where it may reverse chemoresistance or sensitize to other chemotherapeutic agents.

## INTRODUCTION

Evasion of apoptosis is one of the key hallmarks of cancer [1]. Most cancer chemotherapeutic agents kill cancer cells by induction of apoptosis through perturbation of mitochondria and induction of the intrinsic pathway of apoptosis [2, 3]. Major efforts have been made over the last decade to develop small molecule inhibitors of the anti-apoptotic members of the BCL-2 family of proteins, which are highly expressed in some cancers and are known to regulate mitochondrial membrane integrity. Although development of such inhibitors has proved particularly difficult due to the necessity to inhibit protein-protein interactions, some success has been achieved. Most notable is the case of ABT-737 and its orally active analogue, ABT-263 (Navitoclax), both of which inhibit BCL-2, BCL-X_L_ and BCL-w, whereas a related analogue, ABT-199 selectively inhibits BCL-2, and not BCL-X_L_, thus circumventing a dose-limiting thrombocytopenia, associated with BCL-X_L_inhibition [4-10]. Although these inhibitors have recently entered clinical trials for the treatment of various hematological malignancies, the effectiveness of these agents in several cancers is often limited by chemoresistance, which has most commonly been ascribed to high expression levels of other anti-apoptotic BCL-2 family members, particularly MCL-1 [7, 8, 10-15]. Several putative inhibitors of MCL-1 have been reported in the literature, although to our knowledge, none of the compounds appear to be selective or sufficiently potent inhibitors of MCL-1, at least in a cellular context [16].

The lower affinity of commonly available BCL-2 antagonists to bind and inhibit MCL-1 has been attributed to structural differences in the BH3-binding grove of MCL-1 compared with other BCL-2 family members. Moreover, drugs, such as obatoclax and sabutoclax, that have been reported to displace pro-apoptotic BCL-2 family proteins from MCL-1 lack the specificity to induce MCL-1-dependent apoptosis and are more toxic in cellular models [9, 16, 17]. Similarly, stapled peptides designed to specifically bind the hydrophobic groove of MCL-1 and mimic BH3-only members are yet to demonstrate the desired potency in inducing apoptosis *in vivo* [18-20]. In view of the difficulty in designing a specific MCL-1 inhibitor, other approaches are being used in particular to exploit the known short half-life of MCL-1. Thus, cyclin-dependent kinase (CDK) inhibitors, flavopiridol, roscovitine and seliciclib, which transcriptionally suppress MCL-1, and sorafenib, which diminishes MCL-1 translation, show some promise [13, 14, 21]. Likewise, small molecule inhibitors of deubiquitinases, such as USP9X, offer alternative approaches to tackle MCL-1-mediated chemoresistance [22, 23].

In this manuscript, we assess the selectivity and potency of two putative MCL-1 inhibitors that inhibit MCL-1 by distinct mechanisms. One of these inhibitors is marinopyrrole A (maritoclax), which directly binds MCL-1 and targets it for proteasomal degradation in various haematological cancer cells and some melanoma cells [24-26]. In contrast, dinaciclib is a broad-spectrum CDK inhibitor, and has been shown to downregulate MCL-1 levels, most likely due to transcriptional repression [27-29]. In this study, we show that both dinaciclib and maritoclax induce apoptosis in MEFs and non-small cell lung cancer (NSCLC) cell lines. While dinaciclib is much more potent in downregulating MCL-1 levels, MCL-1 loss by maritoclax is relatively modest. The induction of apoptosis in a MCL-1-dependent manner by both compounds is clearly cell-type specific, as both compounds induce apoptosis in MEFs irrespective of MCL-1 status. In addition to driving the proteasomal turnover of MCL-1, maritoclax also alters the structural and functional integrity of mitochondria and leads to the accumulation of mitochondrial ROS.

## RESULTS

### Dinaciclib and maritoclax induce apoptosis in a Bax/Bak- and caspase-9 -dependent manner

**Figure 1:**
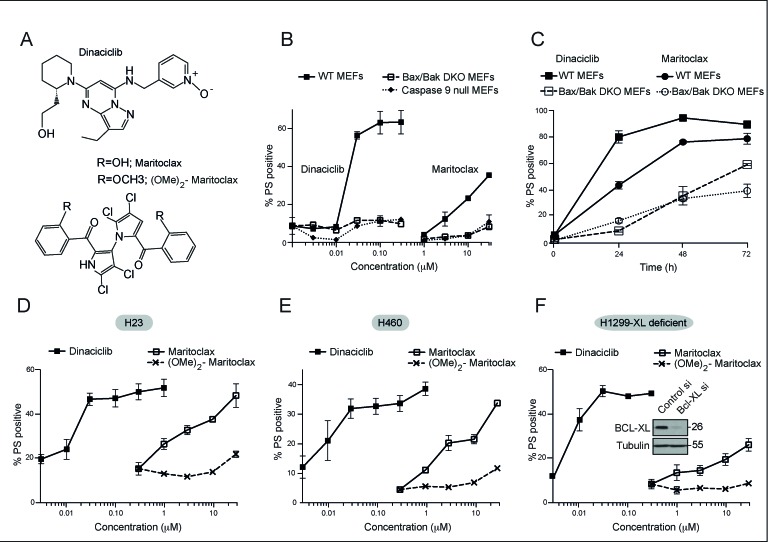
Dinaciclib and maritoclax induce apoptosis in a Bax/Bak- and caspase-9-dependent manner, and in MCL-1-dependent cell lines (**A**) Chemical structures of dinaciclib, maritoclax and the inactive, dimethoxymaritoclax. (**B**) MEFs deficient in either Bax and Bak (DKO) (dashed lines) or caspase-9 (dotted lines) along with their wild type (WT) counterparts (continuous bold lines) were exposed for 24 h to different concentrations of the indicated inhibitors and the extent of apoptosis assessed by phosphatidylserine (PS) externalization. (**C**) WT and DKO MEFs exposed for the indicated times to dinaciclib (100 nM) or maritoclax (10 μM) were assessed for apoptosis by PS externalization. (D-F) Three non-small cell lung cancer, MCL-1-dependent, cell lines, (**D**) H23, (**E**) H460 and (**F**) H1299 cells, reverse-transfected with BCL-X_L_ siRNA for 24 h, were exposed for 24 h to different concentrations of the indicated inhibitors and cell death assessed by PS externalization. The blots in the inset reveal the knockdown efficiency of BCL-X_L_ siRNA. Error bars represent the Mean ± SEM from three independent experiments. In all the graphs, the extent of apoptosis in untreated control cells matched the % apoptosis of the lowest concentration tested for both inhibitors.

Anti-apoptotic members of the BCL-2 family regulate mitochondrial integrity in part by sequestering their pro-apoptotic counterparts, thereby preventing cytochrome *c* release and subsequent activation of caspases in the intrinsic pathway of apoptosis. Small molecule inhibitors of the anti-apoptotic BCL-2 family members have been designed to release the sequestered pro-apoptotic members, which then can induce a Bax/Bak-dependent release of cytochrome *c* and subsequent activation of caspase-9-mediated apoptosis. In this study, we use dinaciclib and maritoclax, two structurally dissimilar compounds, that antagonize MCL-1 activity by distinct mechanisms [24-27, 29, 30]. Substitution of the two side chain hydroxyl groups in maritoclax with methoxy groups results in an inactive variant, dimethoxymaritoclax [31] (Fig. 1A). In MEFs that are either wild type, or deficient in Bax and Bak (DKO) or caspase-9 (caspase-9 null), both dinaciclib and maritoclax induced a concentration-dependent apoptosis in a manner that was completely dependent on Bax/Bak and caspase-9 (Fig. 1B). However, dinaciclib appeared more potent than maritoclax, in inducing apoptosis at nanomolar concentrations, whereas concentrations of maritoclax as high as 3 μM induced only modest levels of cell death (Fig. 1B). The dependence on Bax and Bak to induce apoptosis following maritoclax and dinaciclib did not persist for more than 24 h, as prolonged exposure (72 h) to both maritoclax and dinaciclib resulted in a gradual induction of apoptosis even in DKO cells (Fig. 1C).

### Dinaciclib and maritoclax induce apoptosis in cells that depend on MCL-1 for survival

Since dinaciclib and maritoclax have been shown to target MCL-1 synthesis and degradation respectively, we examined their efficacy in inducing apoptosis in three MCL-1-dependent NSCLC cell lines, H23, H460 and H1299 (Figs. 1D-1F). H23 and H460 are dependent on MCL-1 for survival [15], whereas H1299 is dependent on both MCL-1 and BCL-X_L_ for survival [16]. Therefore, we used RNA interference to silence the expression of BCL-X_L_, thus rendering the H1299 cell line solely MCL-1-dependent. Both dinaciclib and maritoclax caused a concentration-dependent induction of apoptosis in the NSCLC cell lines, whereas dimethoxymaritoclax failed to induce apoptosis, even at 30 μM (Figs. 1D-1F). However as in the MEFs, dinaciclib was significantly more potent than maritoclax at inducing apoptosis (Figs. 1D-1F). Thus the ability to induce apoptosis in several MCL-1-dependent cell lines supported the hypothesis that both dinaciclib and maritoclax targeted MCL-1 and resulted in cell death.

### Dinaciclib decreases MCL-1 expression levels and synergizes with navitoclax in mediating apoptosis

**Figure 2:**
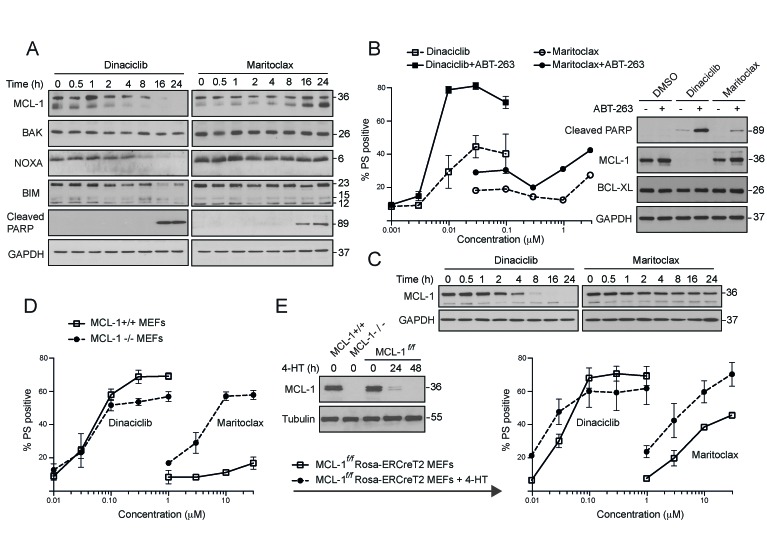
Dinaciclib and maritoclax diminish MCL-1 expression levels and induce MCL-1-dependent apoptosis in a cell-type specific manner (**A**) Whole cell lysates isolated from H460 cells, exposed to dinaciclib (30 nM) or maritoclax (3 μM) for the indicated times, were immunoblotted with the indicated antibodies. (**B**) H1299 cells were exposed to DMSO or ABT-263 (5 μM) for 30 min, followed for a further 24 h by the indicated concentrations of dinaciclib or maritoclax and cell death assessed by PS externalization. Western blots reveal changes in MCL-1 expression and PARP cleavage in H1299 cells, following 24 h of exposure to dinaciclib (30 nM) or maritoclax (3 μM), with or without a 30 min pretreatment of ABT-263 (5 μM). (**C**) Whole cell lysates from H1299 cells exposed to dinaciclib (30 nM) or maritoclax (3 μM) for the indicated times were immunoblotted with the indicated antibodies. (**D**) MEFs deficient in MCL-1 (dashed lines) along with their wild type counterparts (continuous bold lines) were exposed for 24 h to the indicated inhibitors and apoptosis assessed by PS externalization. (**E**) MCL-1^*f/f*^Rosa-ERCreT2 MEFs were initially exposed to DMSO or 4-hydroxytamoxifen (4-HT) (100 nM) to delete endogenous MCL-1. The cells were then exposed for a further 24 h to the indicated inhibitors and apoptosis assessed. Western blots reveal changes in MCL-1 expression in different MEFs, following 4-HT exposure for 0, 24 or 48 h. Error bars represent the Mean ± SEM from three independent experiments. In all the graphs, the extent of apoptosis in untreated control cells matched the % apoptosis of the lowest concentration tested for both inhibitors.

Although both dinaciclib and maritoclax induce apoptosis in MCL-1-addictive cell lines, the mechanisms by which the compounds inhibit MCL-1 are quite distinct. In the MCL-1-dependent H460 cells, dinaciclib caused a significant reduction of MCL-1 protein levels, as early as 2 h and was almost complete at 8 h (Fig. 2A). Dinaciclib also resulted in a time-dependent, near-complete loss of BIM and NOXA, but the loss of BAK was more modest (Fig. 2A). The loss of MCL-1 and its interacting partners preceded the cleavage of the canonical caspase substrate, poly (ADP-ribose) polymerase (PARP) (Fig. 2A). Maritoclax resulted in a much more modest loss of MCL-1, which, at later time points, was not just restored but resulted in protein levels higher than control cells (Fig. 2A). However, maritoclax also resulted in PARP cleavage at 16-24 h (Fig. 2A), suggesting that the early loss of MCL-1 was sufficient to trigger apoptotic events in these cells. Next, we wished to assess if this early loss of MCL-1 could synergize with BCL-2 family inhibitors, such as navitoclax, which inhibit BCL-2, BCL-X_L_ and BCL-w, but not MCL-1, in enhancing apoptosis. For this experiment, we used H1299 cells, which depend on BCL-X_L_ and MCL-1 for survival [16]. Pretreatment of H1299 cells for 30 min with navitoclax greatly enhanced the ability of dinaciclib to induce apoptosis (Fig. 2B). This potentiation of apoptosis was accompanied by the total loss of MCL-1 and significant PARP cleavage (Figs. 2B and 2C). In marked contrast, the extent of apoptosis induction, as evidenced by PARP processing and PS externalization, in cells exposed to navitoclax and maritoclax, was more modest (Figs. 2B and 2C). These observations suggest that a complete loss of MCL-1, as observed with dinaciclib, may be required to observe synergy with navitoclax.

### Dinaciclib and maritoclax can induce apoptosis in a MCL-1-independent manner

Since dinaciclib and maritoclax induced MCL-1-dependent apoptosis in the MCL-1-addictive NSCLC cell lines, we wished to assess if targeting MCL-1 was the predominant mechanism by which these compounds induced apoptosis. For this, we used wild type and MCL-1-deficient MEFs, which do not rely on MCL-1 for survival [32]. Dinaciclib (≤ 100 nM) induced similar levels of apoptosis in both wild type and MCL-1-deficient MEFs, whereas at higher concentrations (> 100 nM), apoptosis occurred in a MCL-1-dependent manner (Fig. 2D). These results suggested that, at least in MEFs, the majority of cell death induced by dinaciclib occurred irrespective of MCL-1 status. In marked contrast, maritoclax induced more cell death in the MCL-1-deficient than wild type MEFs (Fig. 2D), suggesting that although maritoclax may preferentially target MCL-1, it clearly can induce apoptosis through other mechanisms. To avoid incorrect interpretations from possible gene compensation that may have occurred following MCL-1 knockdown, we wished to confirm these results in MCL-1*^f/f^* Rosa-ERCreT2 MEFs, in which endogenous MCL-1 undergoes rapid deletion following exposure to 4-hydroxytamoxifen (4-HT) [32]. Exposure to 4-HT for 24 h was sufficient to deplete MCL-1 levels in these cells, and the response to dinaciclib was fairly similar to our previous data in MCL-1-deficient MEFs (Fig. 2E). However, maritoclax behaved differently in these cells and induced significant cell death even in the presence of MCL-1 (Fig. 2E). Taken together, these data suggest that dinaciclib and maritoclax, despite causing rapid loss of MCL-1, can also induce apoptosis in a MCL-1-independent manner.

### Dinaciclib and maritoclax perturb mitochondria

**Figure 3:**
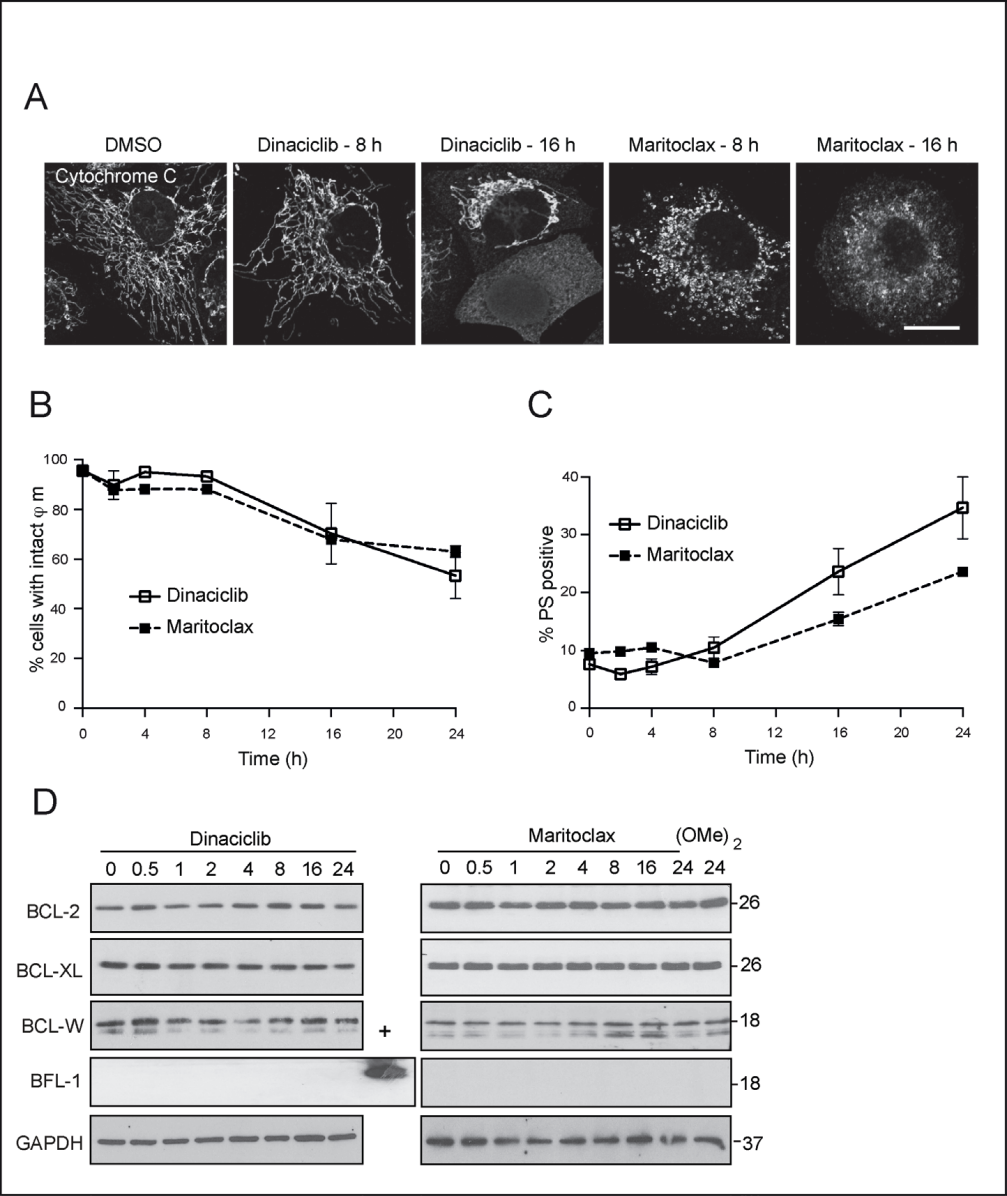
Dinaciclib and maritoclax exert their effects upstream of cytochrome c release and loss in mitochondrial membrane potential (**A**) H460 cells, grown on coverslips, were exposed for 8 or 16 h to dinaciclib (30 nM) or maritoclax (3 μM), stained with the indicated primary and secondary antibodies and subjected to confocal microscopy. Scale bar – 10 μm. (B and C) H460 cells exposed to dinaciclib (30 nM) or maritoclax (3 μM) for the indicated times were stained with (**B**) TMRE to monitor changes in mitochondrial membrane potential (φ_m_) or (**C**) Annexin V-FITC to assess cell death by measuring PS externalization. Error bars represent the Mean ± SEM from three independent experiments. (**D**) Whole cell lysates from H460 cells, exposed to dinaciclib (30 nM), maritoclax (3 μM) or dimethoxymaritoclax (3 μM) for the indicated times, were immunoblotted with the indicated antibodies. Recombinant BFL-1 protein was used as a positive control for the BFL-1 blot.

Although our data in MEFs demonstrated that dinaciclib and maritoclax could induce apoptosis in a MCL-1-independent manner, these compounds exerted apoptosis in a Bax/Bak and caspase-9-dependent manner (Fig. 1B). Moreover, dinaciclib and maritoclax caused a time-dependent release of cytochrome *c* (Fig. 3A), with a concomitant loss in mitochondrial membrane potential (Fig. 3B) and an increase in PS externalization (Fig. 3C). As these observations placed the immediate effects of dinaciclib and maritoclax at the level of mitochondria, we wished to assess if the compounds modulated other anti-apoptotic members of the BCL-2 family, which may in turn result in apoptosis. In H460 cells, none of the anti-apoptotic members such as BCL-2, BCL-X_L_ or BCL-w were downregulated following either dinaciclib or maritoclax (Fig. 3D). Furthermore, these cells did not express detectable levels of BFL-1 (Fig. 3D). Taken together, our data revealed that maritoclax and dinaciclib may inhibit MCL-1 and induce apoptosis in a cell-type specific manner.

### Maritoclax, but not dinaciclib, results in extensive mitochondrial fragmentation

**Figure 4:**
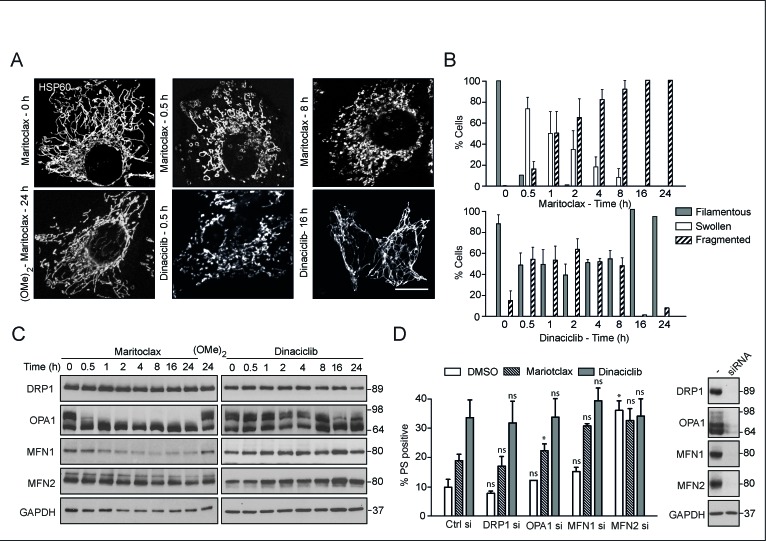
Maritoclax, and dinaciclib induce marked mitochondrial structural changes, which may contribute to apoptosis (**A**) H460 cells, grown on coverslips, were exposed for different times to dinaciclib (30 nM), maritoclax (3 μM) or dimethoxymaritoclax (3 μM), stained with antibody against HSP60 and subjected to confocal microscopy. Scale bar – 10 μm. (**B**) At least 200 cells from five different fields were quantified for changes in mitochondrial morphology (categorised as filamentous, swollen or fragmented) and a graph plotted from three independent replicates. Error bars represent the Mean ± SEM. (**C**) Whole cell lysates from H460 cells, exposed to of dinaciclib (30 nM), maritoclax (3 μM) or dimethoxymaritoclax (3 μM) for the indicated times, were immunoblotted with the indicated antibodies. (**D**) H460 cells, reverse-transfected with the indicated siRNA oligoduplexes for 48 h, were exposed for a further 24 h to dinaciclib (30 nM) or maritoclax (3 μM), and cell death assessed by PS externalization. Error bars represent the Mean ± SEM from three independent experiments. Western blots show the knockdown efficiency of the individual siRNAs. Statistical analysis was conducted using a paired t-test (*P < 0.05, ns – not significant, if *P* > 0.05).

Although dinaciclib and maritoclax caused a time-dependent release of cytochrome *c* (Fig. 3A), our data suggested that these compounds might alter mitochondrial structure and/or function, in an analogous manner to other putative MCL-1 inhibitors [33]. Exposure to maritoclax resulted in rapid mitochondrial swelling, which subsequently lead to extensive mitochondrial fragmentation (Figs. 4A and 4B). Exposure to dinaciclib also resulted in mitochondrial structural changes, although these effects were more subtle (Figs. 4A and 4B). Maritoclax-mediated mitochondrial structural changes accompanied a time-dependent loss both in MFN1 and also in the high molecular weight isoforms of OPA1 but did not alter DRP1 levels, suggesting a loss in mitochondrial fusion (Fig. 4C). Exposure to dinaciclib did not cause similar changes in the expression levels of mitochondrial fission-fusion proteins (Fig. 4C). Previously, we speculated that MCL-1 mediated changes in mitochondrial membrane dynamics could possibly exhibit a cross-talk in the cell death pathway induced by MCL-1 inhibitors [33]. To test our hypothesis, we silenced the expression levels of different mitochondrial fission and fusion proteins, and assessed the extent of cell death following exposure to either maritoclax or dinaciclib. Downregulation of DRP1, OPA1 or MFN1 failed to exhibit any positive influence on maritoclax or dinaciclib-mediated cell death (Fig. 4D), whereas silencing of MFN2 was toxic even in the absence of the inhibitors and no additive/synergistic effect was observed in combination with either dinaciclib or maritoclax (Fig. 4D). These results suggest that MCL-1 possibly regulates mitochondrial fusion dynamics and apoptosis by independent mechanisms, in agreement with previous reports [32].

### Maritoclax but not dinaciclib induces mitochondrial ROS

**Figure 5:**
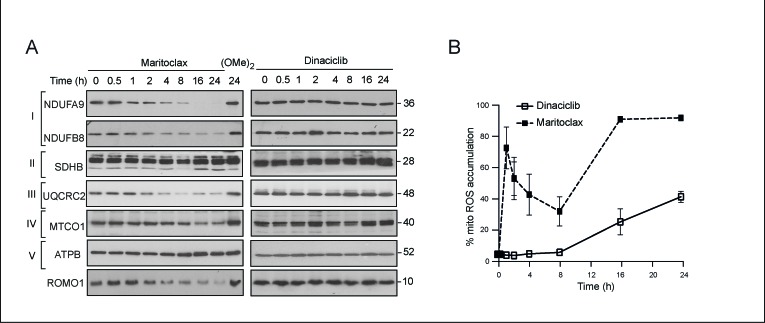
Maritoclax induces a loss of different components of the electron transport chain and an accumulation of mitochondrial ROS (**A**) Whole cell lysates of H460 cells, exposed to dinaciclib (30 nM), maritoclax (3 μM) or dimethoxymaritoclax (3 μM) for the indicated times, were immunoblotted with the indicated antibodies. (**B**) H460 cells, exposed to dinaciclib (30 nM) or maritoclax (3 μM) for the indicated times, were stained with MitosoxRed to monitor the accumulation of mitochondrial reactive oxygen species (ROS). Error bars represent the Mean ± SEM from three independent experiments.

In addition to its proposed role in regulating mitochondrial fusion, MCL-1 is also proposed to regulate mitochondrial homeostasis [32]. We therefore hypothesized that the dramatic changes in mitochondrial ultrastructure should also disrupt mitochondrial function. Maritoclax, but not dinaciclib, resulted in a significant time-dependent loss of certain components of complexes I and III of the electron transport chain (Fig. 5A). Both these complexes have been implicated in the generation of ROS [34]. Furthermore, maritoclax but not dinaciclib, also resulted in a similar depletion of ROMO1 (Fig. 5A), which was recently recognized as a sensor for mitochondrial ROS [35]. Consistent with these observations, maritoclax resulted in a rapid accumulation of mitochondrial ROS, as assessed by mitosoxRed staining (Fig. 5B). In contrast, exposure to dinaciclib caused little or no build-up of mitochondrial ROS at the initial time points and an increase was only observed at later times (16-24 h) (Fig. 5B).

### Maritoclax-mediated accumulation of mitochondrial ROS, but not membrane fragmentation, occurs in a Bax/Bak-dependent manner

**Figure 6:**
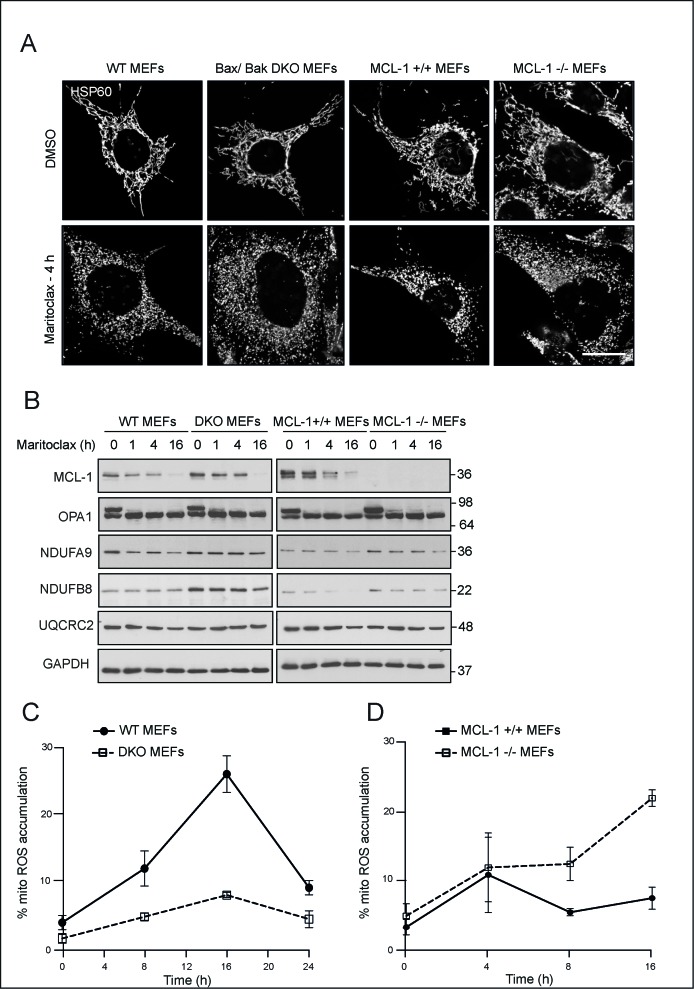
Maritoclax-mediated mitochondrial effects occur independent of Bax/Bak- and MCL-1 (**A**) MEFs deficient in either Bax/Bak (DKO) or MCL-1 along with their wild type counterparts were grown on coverslips, exposed for 4 h to maritoclax (10 μM), stained with antibody against HSP60 and subjected to confocal microscopy. Scale bar – 10 μm. (**B**) MEFs deficient in either Bax/Bak (DKO) or MCL-1 along with their wild type counterparts exposed to maritoclax (10 μM) for the indicated times, were immunoblotted with the indicated antibodies. (C and D) MEFs deficient in either Bax/Bak (DKO) (**C**) or MCL-1 (**D**) along with their wild type counterparts were exposed to maritoclax (10 μM) for the indicated times and mitochondrial accumulation of ROS assessed. Error bars represent the Mean ± SEM from three independent experiments.

Although maritoclax-mediated effects on mitochondrial fragmentation and ROS accumulation were consistent with the purported roles of MCL-1, we wished to assess whether maritoclax induced such changes in a Bax/Bak- and MCL-1- dependent manner. Maritoclax induced extensive mitochondrial fragmentation in both DKO and MCL-1 null MEFs, to a similar extent as their wild type counterparts (Fig. 6A), thus suggesting that maritoclax-induced mitochondrial fragmentation occurred irrespective of Bax/Bak and MCL-1 status. In agreement with the microscopic observation of mitochondrial fragmentation, maritoclax resulted in a loss of high molecular weight isoforms of OPA1 in cells that lacked either Bax/Bak or MCL-1 (Fig. 6B). However, the loss of ETC components observed following maritoclax in wild type cells, seem to diminish in the DKO cells, suggesting an involvement of Bax/Bak in accelerating maritoclax-mediated loss of ETC components (Fig. 6B). Maritoclax-mediated rapid accumulation of mitochondrial ROS also occurred in a Bax/Bak-dependent manner, which raised the possibility that the mitochondrial ROS were responsible for the loss of the ETC components (Fig. 6C). However, a similar extent of protection either from the loss of ETC components or mitochondrial ROS accumulation was not observed in the MCL-1 null MEFs, thus ruling out MCL-1 in these events (Figs. 6B and 6D). Moreover, maritoclax induced a greater accumulation of mitochondrial ROS in cells lacking MCL-1 (Fig. 6D), similar to the cell death effects observed in these cells (Fig. 2D).

### MitoQ does not prevent maritoclax-mediated mitochondrial effects

**Figure 7:**
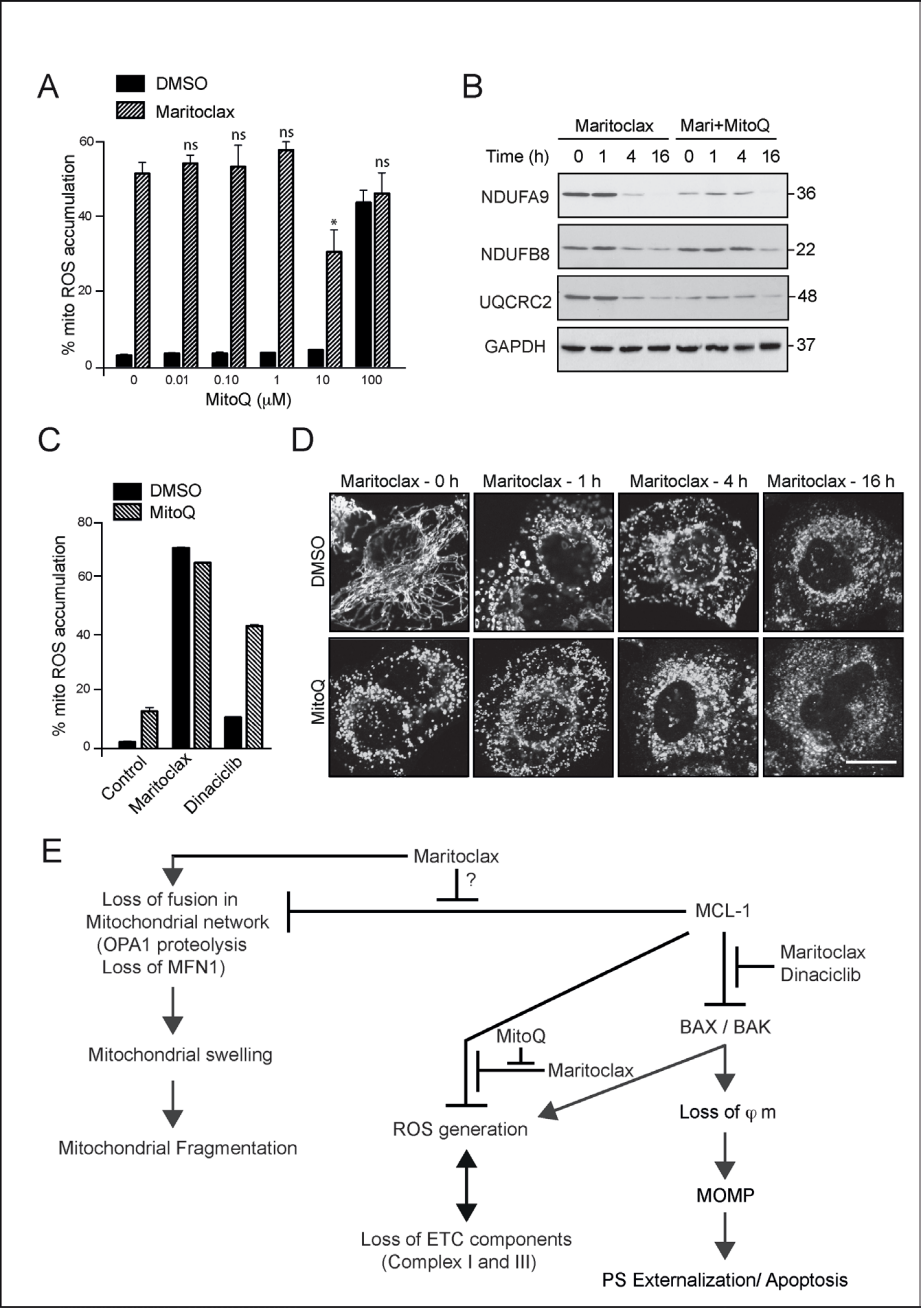
Maritoclax-mediated mitochondrial effects are partially rescued by MitoQ (**A**) H460 cells were exposed to different concentrations of MitoQ for 4 h, followed by maritoclax (3 μM) for a further 2 h, and mitochondrial accumulation of ROS assessed. Statistical analysis was conducted using a paired t-test (*P < 0.05, ns – not significant, if *P* > 0.05). (**B**) Whole cell lysates from H460 cells, exposed to MitoQ (10 μM) for 4 h followed by maritoclax (3 μM) for the indicated times, were immunoblotted with the indicated antibodies. (**C**) H460 cells exposed to MitoQ (10 μM) for 4 h, followed by either Maritoclax (3 μM) or dinaciclib (30 nM) for a further 20 h were stained with MitosoxRed to monitor changes in mitochondrial ROS accumulation. Error bars represent the Mean ± SEM from three independent experiments. (**D**) H460 cells, grown on coverslips, were exposed for 4 h to MitoQ (10 μM), followed by maritoclax (3 μM) for the indicated times. The cells were then immunostained with HSP60 antibody and subjected to confocal microscopy. Scale bar – 10 μm. (**E**) Scheme for proposed mechanism of action of dinaciclib and maritoclax. Dinaciclib and maritoclax interfere with the anti-apoptotic effects of MCL-1, in a Bax/Bak and caspase-9-dependent manner, by causing the loss of mitochondrial membrane potential (φm) and activation of mitochondrial outer membrane permeabilization (MOMP), resulting in apoptosis. Maritoclax, but not dinaciclib, interfered with other functions of MCL-1, including the postulated regulation of mitochondrial fusion, accompanied by enhanced OPA1 proteolysis and loss in MFN1, all of which resulted in mitochondrial structural changes that initially manifested as swelling and subsequently resulted in extensive mitochondrial fragmentation. However, maritoclax-mediated changes in mitochondrial structure also occurred in cells lacking MCL-1, suggesting the involvement of MCL-1-independent mechanisms in these processes. Maritoclax also resulted in ROS generation in mitochondria, which accompanied a loss in several components of the electron transport chain, in a Bax/Bak-dependent but a MCL-1-independent manner, thus placing Bax/Bak upstream to mitochondrial ROS induction and the loss of ETC components.

To assess if maritoclax-mediated accumulation of mitochondrial ROS preceded the loss in complexes I and III, we attempted to scavenge mitochondrial ROS using MitoQ [36]. Pretreatment with MitoQ (10 μM) for 4 hour was necessary to partially rescue the ROS (Fig. 7A), which in turn partially prevented the loss of NDUFB8 component of complex I (Fig. 7B). No such protection was observed against the loss of NDUFA9 and UQCRC2, as MitoQ appeared to hasten the loss of these components, both in the presence and absence of maritoclax (Fig. 7B). Moreover, the protective effect of MitoQ against the loss of these components was short-lived, as it failed to sustain its protective effects at later times (16 h), both with respect to preventing the loss of complexes and in rescuing maritoclax-mediated ROS accumulation (Fig. 7B & 7C). In fact, MitoQ appeared to enhance the extent of dinaciclib-mediated ROS accumulation at 16 h, suggesting that prolonged exposure to high concentrations of MitoQ could not mimic the protective responses, observed at earlier time points (Fig. 7C). The inability of MitoQ to sustain its protective effects precluded its valid use to examine the role if any of ROS in maritoclax-induced apoptosis. Although MitoQ failed to prevent ROS accumulation at later times, it was still effective in partially rescuing the effects at 2 h of exposure to maritoclax (Fig. 7A). Therefore, we wished to assess if scavenging maritoclax-mediated ROS accumulation could reverse maritoclax-mediated effects on mitochondrial structure. Pretreatment with MitoQ not only failed to prevent maritoclax-mediated mitochondrial swelling and fragmentation, but also resulted in excessive mitochondrial fragmentation, on its own, independent of maritoclax (Fig. 7D).

## DISCUSSION

Enhanced expression levels of anti-apoptotic BCL-2 family proteins in several cancers make them promising targets for drug therapy. Small molecule inhibitors targeting specific members of the BCL-2 family, such as navitoclax (BCL-2/BCL-X_L_/BCL-w) and ABT-199 (BCL-2 specific) are currently in clinical trials. These inhibitors are however ineffective against MCL-1, which is commonly amplified in human tumours and associated with tumour relapse and chemoresistance. Although several reports claiming the discovery of novel inhibitors of MCL-1 have been published, to our knowledge, none of these inhibitors have demonstrated significant potency and/or specificity in a cellular context [16]. In this study, we evaluate the potency and specificity of dinaciclib and maritoclax, both of which exert MCL-1 inhibition *via* distinct mechanisms [24-26, 29, 30]. Our data indicate that both maritoclax and dinaciclib efficiently downregulate the expression levels of MCL-1 and induce death in a Bax/Bak- and caspase-9-dependent manner (Figs. 1 and 2).

Dinaciclib is a broad-spectrum CDK inhibitor and is currently in clinical trials to treat several cancers [27, 28, 37-41]. Owing to its ability to inhibit multiple CDKs, dinaciclib could attenuate the expression levels of several short-lived proteins, such as MCL-1, which may in turn trigger the apoptotic cascade in several cancers. Apoptosis mediated by BCL-2 family antagonists is believed to result in release of pro-apoptotic members, such as BIM and NOXA, which subsequently induce apoptosis. In our experiments, dinaciclib decreased the expression levels not only of MCL-1 but also of BIM and NOXA (Fig. 2). Thus dinaciclib appears to mediate apoptosis in a BIM- and NOXA-independent but a Bax/Bak-dependent manner. Moreover, dinaciclib induced apoptosis irrespective of MCL-1 expression levels (Figs. 2D and 2E), demonstrating that the apoptotic effects of dinaciclib can occur in a MCL-1-dependent and independent manner and cannot be attributed solely to MCL-1 downregulation.

Although dinaciclib is clearly not a specific MCL-1 inhibitor, its ability to rapidly downregulate MCL-1 in several cell types, may clearly be beneficial in many clinical settings, where it may reverse chemoresistance or sensitize to other chemotherapeutic agents, such as ABT-199 or navitoclax. Furthermore, the ability of dinaciclib to inhibit the transcription of other short-lived proteins, which may also regulate cell death, could be beneficial. In this regard, dinaciclib is currently in a number of early clinical trials either alone or in combination with various chemotherapeutic agents including capecitabine, erlotinib, carboplatin and bortezomib to evaluate its efficacy in various malignancies, including non-small cell lung cancer, triple negative breast cancer, pancreatic cancer, melanoma, multiple myeloma and refractory chronic lymphocytic leukemia [42].

Unlike dinaciclib, maritoclax has been shown to specifically target MCL-1 for proteasomal degradation in experimental models of melanoma and various haematological malignancies [24-26]. In our studies, maritoclax was not as potent as dinaciclib in downregulating MCL-1 and inducing apoptosis (Figs. 1 and 2). Moreover, the loss of MCL-1 following maritoclax was transient in H460 cells, as levels of MCL-1 were even elevated at later times through an unknown mechanism (Fig. 2A). In contrast, a time-dependent reduction of Mcl-1 was observed in H1299 cells (Fig. 2C) and MEFs (Fig. 6B) treated with maritoclax. Maritoclax induced more cell death in MEFs deficient in MCL-1 compared with the wild type MEFs (Fig. 2C), possibly due to its sequestration by MCL-1 thereby resulting in less free maritoclax to induce apoptosis by other mechanisms. These results suggest that the ability of maritoclax to induce Mcl-1-dependent apoptosis may be cell type dependent, in agreement with a recent report [43].

In addition to its conventional role in apoptosis, MCL-1 has been implicated in other cellular functions ranging from the regulation of ER and mitochondrial membrane dynamics to mitochondrial bioenergetics [33, 44-47]. Inhibitors of MCL-1 have resulted in extensive mitochondrial fragmentation and cristae remodelling, which have been attributed to a loss of fusion rather than enhanced mitochondrial fission [9, 33]. Furthermore, accumulation of mitochondrial ROS, a significant decrease in ATP production and a near complete loss of the components of electron transport chain have all been associated with MCL-1 inhibition [32, 33]. Consistent with these reports, maritoclax, but not dinaciclib interfered with mitochondrial fusion and function (Figs. 4 and 5), which may be due to the distinct mechanisms by which dinaciclib and maritoclax downregulate MCL-1. However, maritoclax induced extensive mitochondrial fragmentation and OPA1 proteolysis even in cells lacking MCL-1, suggesting that maritoclax-induced mitochondrial fragmentation occurred irrespective of MCL-1 status (Fig. 6). Similarly, the loss of ETC components and accumulation of mitochondrial ROS also occurred in a MCL-1-independent manner, thus confirming that maritoclax-mediated mitochondrial changes may not be due entirely to MCL-1 downregulation. Nevertheless, the loss of ETC components and the rapid accumulation of mitochondrial ROS, following maritoclax, occurred in a Bax/Bak-dependent manner, thus placing Bax/Bak upstream to mitochondrial ROS induction and the loss of ETC components.

Moreover, attempts to rescue mitochondrial ROS with several ROS scavengers, including SS31, N-acetylcysteine, Tiron, Mn(III)TBAP and EUK134 were ineffective, and only a partial protection was achieved by MitoQ (Fig. 7 and data not shown). Despite the partial rescue, MitoQ failed to prevent mitochondrial structural and functional changes (Fig. 7). In fact, MitoQ alone, resulted in significant mitochondrial fragmentation suggesting other off-target effects (Fig. 7D). Therefore presently, it is difficult to assess if maritoclax-mediated increase in mitochondrial ROS and/or a loss in mitochondrial membrane fusion could be directly linked to MCL-1 regulated death. Further insights into the mechanisms by which MCL-1 regulates these disparate functions are required for us to definitely delineate a potential crosstalk among these responses (Fig. 7E).

## MATERIALS AND METHODS

### Cell culture

Wild type and Bax/Bak DKO MEFs from Dr. A. Strasser (WEHI, Melbourne, Australia) and caspase-9 null MEFs from Prof. T. Mak (University of Toronto, Toronto, Canada), MCL-1 deficient and MCL-1*^f/f^*Rosa-ERCreT2 MEFs from Prof. J. Opferman (St Jude Children's Research Hospital, Memphis, TN, USA) were cultured in DMEM medium supplemented with 10% fetal calf serum (FCS) and 5 mM L-glutamine (all from Life Technologies Inc, Paisley, UK). NSCLC cell lines, H23, H460 and H1299, from ATCC (Middlesex, UK) were cultured in RPMI 1640 medium supplemented with 10% FCS and 5 mM L-glutamine.

### Reagents

Maritoclax (marinopyrrole A) and dimethoxymaritoclax were synthesized with purity >99% as previously described [31]. Dinaciclib was obtained from Stratech Scientific Ltd (Suffolk, UK). ABT-263 was obtained from Selleck Chemicals Co. (Houston, TX, USA). MitoQ was a kind gift from Prof. M. Jackson, University of Liverpool, UK. Antibodies against BIM, cleaved PARP, BCL-X_L_, BCL-w, MFN1 and MFN2 from Cell Signalling Technology Inc (Danvers, MA, USA), BCL-2 from Dako (Ely, UK), BFL-1 (a kind gift from Prof. J. Borst, The Netherlands Cancer Institute, Amsterdam, The Netherlands), ROMO1 from OriGene (Rockville, MD, USA), cytochrome *c*, DRP1 and OPA1 from BD biosciences (Oxford, UK), SDHB, UQCRC2, MTCO1, and ATPB from Abcam (Cambridge, UK), NOXA and tubulin from Calbiochem (Nottingham, UK), MCL-1, GAPDH, NDUFA9 and NDUFB8 from Santa Cruz Biotechnology (Santa Cruz, CA, USA) and BAK from Millipore (Watford, UK) were used. MitosoxRed was purchased from Molecular Probes, Inc. (Eugene, OR, USA). All other reagents, unless mentioned otherwise, were from Sigma-Aldrich Co. (St. Louis, MO, USA).

### siRNA knockdowns and western blotting

Cells were reverse-transfected with 10 nM of BCL-X_L_ (ID No. s1920), DRP-1 (ID No. s19560), OPA1 (ID No. s9850), MFN1 (ID No. s31220) or MFN2 (pool of siRNAs) oligoduplexes from either Life Technologies Inc. or Santa Cruz Biotechnology using Interferin Reagent (Polyplus transfection Inc, NY, USA), according to the manufacturer's protocol and processed 48 h after transfection. Western blots were carried out according to standard protocols [9].

### Flow cytometry and microscopy

Loss in mitochondrial membrane potential (φm), phosphatidylserine (PS) externalization and mitochondrial ROS accumulation were assessed as described previously [33, 48]. For immunofluorescent staining, cells grown on coverslips were fixed with 4% (v/v) paraformaldehyde, permeabilized with 0.5% (v/v) Triton X-100 in PBS, followed by incubations with antibodies and analyzed, as previously described [16].
